# Supplementary material for: Comprehensive Bioinformatics Analysis the circRNAs of Viral Infection Associated Pathway in HepG2 Expressing ORF3 of Genotype IV Swine Hepatitis E Virus
Source: Microorganisms. 2025 Nov 22;13(12):2654. doi: 10.3390/microorganisms13122654 (PMC12734725; doi:10.3390/microorganisms13122654)
Supplement: Supplementary file 1 [file microorganisms-13-02654-s001.zip › microorganisms-3982438-supplementary.pdf]

Table S1. miRNA predicted by differential expression of mRNA.

mature_mirna_id	target_symbol
hsa-miR-19a-3p	SOCS3
hsa-miR-19b-3p	SOCS3
hsa-miR-17-5p	EGR2
hsa-miR-203a-3p	SOCS3
hsa-miR-130a-3p	HSPA8
hsa-miR-30e-5p	SOCS3
hsa-miR-449a	HSPA1B
hsa-miR-455-5p	SOCS3
hsa-miR-20a-5p	EGR2
hsa-miR-26a-5p	HSPA8
hsa-miR-26b-5p	HSPA8
hsa-miR-30a-5p	SOCS3
hsa-miR-106a-5p	EGR2
hsa-miR-30c-5p	SOCS3
hsa-miR-30d-5p	SOCS3
hsa-miR-139-5p	CXCR4
hsa-miR-34a-5p	HSPA1B
hsa-miR-30b-5p	SOCS3
hsa-miR-106b-5p	EGR2
hsa-miR-301a-3p	HSPA8
hsa-miR-130b-3p	HSPA8
hsa-miR-374a-5p	CCL2
hsa-miR-20b-5p	EGR2
hsa-miR-519d-3p	EGR2
hsa-miR-454-3p	HSPA8
hsa-miR-301b-3p	HSPA8
hsa-miR-17-5p	HSPA8
hsa-miR-20a-5p	HSPA8
hsa-miR-106a-5p	HSPA8
hsa-miR-181a-5p	ZNF527
hsa-miR-181b-5p	ZNF527
hsa-miR-181c-5p	ZNF527
hsa-miR-204-5p	CXCR4
hsa-miR-1-3p	NOTCH3
hsa-miR-124-3p	EGR2
hsa-miR-140-5p	EGR2
hsa-miR-142-3p	EGR2
hsa-miR-191-5p	EGR1
hsa-miR-150-5p	EGR2
hsa-miR-206	NOTCH3
hsa-miR-106b-5p	HSPA8
hsa-miR-34c-5p	HSPA1B
hsa-miR-361-5p	ZNF460
hsa-miR-20b-5p	HSPA8
hsa-miR-202-3p	LAMA1

hsa-miR-181d-5p	ZNF527
hsa-miR-524-5p	EGR2
hsa-miR-506-3p	EGR2
hsa-miR-532-5p	CXCL2
hsa-miR-374b-5p	CCL2
hsa-miR-1297	HSPA8

Table S2.Seven identified mRNAs and their involved viral infection pathways and related sequencing data

gene_id	gene_name	GO pathway	KEGG pathway	log2 (fc)	pval	regulation	significant
ENSG00000121966	CXCR4	GO:0001618 (virus receptor activity)<br>GO:0009615 (response to virus)<br>GO:0016032 (viral process)<br>GO:0006915 (apoptotic process)<br>GO:0006954 (inflammatory response)<br>GO:0006955 (immune response)<br>GO:0007165 (signal transduction)<br>GO:0019064 (fusion of virus membrane with host plasma membrane)<br>GO:0050792 (regulation of viral process)	04062 (Chemokine signaling pathway)	2.06	<0.01	up	yes
ENSG00000184557	SOCS3	GO:0007165 (signal transduction)<br>GO:0007259 (receptor signaling pathway via JAK-STAT)<br>GO:0043066 (negative regulation of apoptotic process)	04630 (Jak-STAT signaling pathway)<br>04668 (TNF signaling pathway)<br>05160 (Hepatitis C)	1.56	<0.01	up	yes
ENSG00000122877	EGR2	No viral infection-related GO terms were enriched for this gene.	05161 (Hepatitis B)	1.40	<0.01	up	yes
ENSG00000204388	HSPA1B	GO:0001618 (virus receptor activity)<br>GO:0016032 (viral process)<br>GO:0046718 (viral entry into host cell)<br>GO:0043066 (negative regulation of apoptotic	04010 (MAPK signaling pathway)<br>04612 (Antigen processing and presentation)	-1.85	<0.01	down	yes

gene_id	gene_name	GO pathway	KEGG pathway	log2 (fc)	pval	regulation	significant
		process)					
ENSG00000109971	HSPA8	GO:0016032 (viral process)	04010 (MAPK signaling pathway) 04612 (Antigen processing and presentation)	-1.06	<0.01	down	yes
ENSG00000074181	NOTCH3	No viral infection-related GO terms were enriched for this gene.	04658 (Th1 and Th2 cell differentiation)	1.78	<0.01	up	yes
ENSG00000189164	ZNF527	No viral infection-related GO terms were enriched for this gene.	05168(Herpes simplex virus 1 infection)	-1.07	<0.01	down	yes

Table S3. Screen out the relevant circRNA-miRNA-mRNA.

circRNA	miRNA	mRNA
ciRNA194	hsa-miR-139-5p	CXCR4
ciRNA194	hsa-miR-30a-5p	SOCS3
ciRNA194	hsa-miR-30b-5p	SOCS3
ciRNA194	hsa-miR-30c-5p	SOCS3
ciRNA194	hsa-miR-30d-5p	SOCS3
ciRNA194	hsa-miR-34a-5p	HSPA1B
ciRNA203	hsa-miR-1-3p	NOTCH3
ciRNA203	hsa-miR-106a-5p	EGR2
ciRNA203	hsa-miR-106a-5p	HSPA8
ciRNA203	hsa-miR-124-3p	EGR2
ciRNA203	hsa-miR-130a-3p	HSPA8
ciRNA203	hsa-miR-139-5p	CXCR4
ciRNA203	hsa-miR-140-5p	EGR2
ciRNA203	hsa-miR-17-5p	EGR2
ciRNA203	hsa-miR-17-5p	HSPA8
ciRNA203	hsa-miR-181a-5p	ZNF527
ciRNA203	hsa-miR-181b-5p	ZNF527
ciRNA203	hsa-miR-181c-5p	ZNF527
ciRNA203	hsa-miR-203a-3p	SOCS3
ciRNA203	hsa-miR-204-5p	CXCR4
ciRNA203	hsa-miR-20a-5p	EGR2
ciRNA203	hsa-miR-20a-5p	HSPA8
ciRNA203	hsa-miR-26a-5p	HSPA8
ciRNA203	hsa-miR-26b-5p	HSPA8
ciRNA203	hsa-miR-34a-5p	HSPA1B
ciRNA203	hsa-miR-93-5p	EGR2
ciRNA203	hsa-miR-93-5p	HSPA8
circRNA14048	hsa-miR-26a-5p	HSPA8

circRNA14048	hsa-miR-26b-5p	HSPA8
circRNA14720	hsa-miR-140-5p	EGR2
circRNA14720	hsa-miR-34a-5p	HSPA1B
circRNA14799	hsa-miR-140-5p	EGR2
circRNA14900	hsa-miR-139-5p	CXCR4
circRNA14936	hsa-miR-106a-5p	EGR2
circRNA14936	hsa-miR-106a-5p	HSPA8
circRNA14936	hsa-miR-17-5p	EGR2
circRNA14936	hsa-miR-17-5p	HSPA8
circRNA14936	hsa-miR-20a-5p	EGR2
circRNA14936	hsa-miR-20a-5p	HSPA8
circRNA14936	hsa-miR-34a-5p	HSPA1B
circRNA14936	hsa-miR-93-5p	EGR2
circRNA14936	hsa-miR-93-5p	HSPA8
circRNA17257	hsa-miR-124-3p	EGR2
circRNA17257	hsa-miR-139-5p	CXCR4
circRNA17257	hsa-miR-204-5p	CXCR4
circRNA5112	hsa-miR-203a-3p	SOCS3
circRNA5119	hsa-miR-181a-5p	ZNF527
circRNA5119	hsa-miR-181b-5p	ZNF527
circRNA5119	hsa-miR-181c-5p	ZNF527
circRNA5199	hsa-miR-1-3p	NOTCH3
circRNA5510	hsa-miR-1-3p	NOTCH3
circRNA5510	hsa-miR-130a-3p	HSPA8
circRNA5510	hsa-miR-181a-5p	ZNF527
circRNA5510	hsa-miR-181b-5p	ZNF527
circRNA5510	hsa-miR-181c-5p	ZNF527
circRNA5510	hsa-miR-26a-5p	HSPA8
circRNA5510	hsa-miR-26b-5p	HSPA8
circRNA5510	hsa-miR-34a-5p	HSPA1B
circRNA5562	hsa-miR-1-3p	NOTCH3
circRNA5562	hsa-miR-106a-5p	EGR2
circRNA5562	hsa-miR-106a-5p	HSPA8
circRNA5562	hsa-miR-124-3p	EGR2
circRNA5562	hsa-miR-139-5p	CXCR4
circRNA5562	hsa-miR-140-5p	EGR2
circRNA5562	hsa-miR-17-5p	EGR2
circRNA5562	hsa-miR-17-5p	HSPA8
circRNA5562	hsa-miR-19a-3p	SOCS3
circRNA5562	hsa-miR-19b-3p	SOCS3
circRNA5562	hsa-miR-203a-3p	SOCS3
circRNA5562	hsa-miR-20a-5p	EGR2
circRNA5562	hsa-miR-20a-5p	HSPA8
circRNA5562	hsa-miR-26a-5p	HSPA8
circRNA5562	hsa-miR-26b-5p	HSPA8
circRNA5562	hsa-miR-30b-5p	SOCS3
circRNA5562	hsa-miR-30c-5p	SOCS3
circRNA5562	hsa-miR-93-5p	EGR2
circRNA5562	hsa-miR-93-5p	HSPA8

circRNA5591	hsa-miR-1-3p	NOTCH3
circRNA5591	hsa-miR-106a-5p	EGR2
circRNA5591	hsa-miR-106a-5p	HSPA8
circRNA5591	hsa-miR-124-3p	EGR2
circRNA5591	hsa-miR-130a-3p	HSPA8
circRNA5591	hsa-miR-139-5p	CXCR4
circRNA5591	hsa-miR-140-5p	EGR2
circRNA5591	hsa-miR-17-5p	EGR2
circRNA5591	hsa-miR-17-5p	HSPA8
circRNA5591	hsa-miR-181a-5p	ZNF527
circRNA5591	hsa-miR-181b-5p	ZNF527
circRNA5591	hsa-miR-181c-5p	ZNF527
circRNA5591	hsa-miR-19a-3p	SOCS3
circRNA5591	hsa-miR-19b-3p	SOCS3
circRNA5591	hsa-miR-203a-3p	SOCS3
circRNA5591	hsa-miR-204-5p	CXCR4
circRNA5591	hsa-miR-20a-5p	EGR2
circRNA5591	hsa-miR-20a-5p	HSPA8
circRNA5591	hsa-miR-26a-5p	HSPA8
circRNA5591	hsa-miR-26b-5p	HSPA8
circRNA5591	hsa-miR-30c-5p	SOCS3
circRNA5591	hsa-miR-34a-5p	HSPA1B
circRNA5591	hsa-miR-93-5p	EGR2
circRNA5591	hsa-miR-93-5p	HSPA8
circRNA5617	hsa-miR-26a-5p	HSPA8
circRNA5617	hsa-miR-26b-5p	HSPA8
circRNA5619	hsa-miR-1-3p	NOTCH3
circRNA5619	hsa-miR-106a-5p	EGR2
circRNA5619	hsa-miR-106a-5p	HSPA8
circRNA5619	hsa-miR-130a-3p	HSPA8
circRNA5619	hsa-miR-139-5p	CXCR4
circRNA5619	hsa-miR-140-5p	EGR2
circRNA5619	hsa-miR-17-5p	EGR2
circRNA5619	hsa-miR-17-5p	HSPA8
circRNA5619	hsa-miR-181a-5p	ZNF527
circRNA5619	hsa-miR-181b-5p	ZNF527
circRNA5619	hsa-miR-181c-5p	ZNF527
circRNA5619	hsa-miR-203a-3p	SOCS3
circRNA5619	hsa-miR-204-5p	CXCR4
circRNA5619	hsa-miR-20a-5p	EGR2
circRNA5619	hsa-miR-20a-5p	HSPA8
circRNA5619	hsa-miR-26a-5p	HSPA8
circRNA5619	hsa-miR-26b-5p	HSPA8
circRNA5619	hsa-miR-30a-5p	SOCS3
circRNA5619	hsa-miR-30b-5p	SOCS3
circRNA5619	hsa-miR-30c-5p	SOCS3
circRNA5619	hsa-miR-30d-5p	SOCS3
circRNA5619	hsa-miR-93-5p	EGR2
circRNA5619	hsa-miR-93-5p	HSPA8

circRNA8848	hsa-miR-106a-5p	EGR2
circRNA8848	hsa-miR-106a-5p	HSPA8
circRNA8848	hsa-miR-130a-3p	HSPA8
circRNA8848	hsa-miR-139-5p	CXCR4
circRNA8848	hsa-miR-17-5p	EGR2
circRNA8848	hsa-miR-17-5p	HSPA8
circRNA8848	hsa-miR-204-5p	CXCR4
circRNA8848	hsa-miR-20a-5p	EGR2
circRNA8848	hsa-miR-20a-5p	HSPA8
circRNA8848	hsa-miR-93-5p	EGR2
circRNA9061	hsa-miR-124-3p	EGR2
hsa_circ_0000563	hsa-miR-130a-3p	HSPA8
hsa_circ_0000563	hsa-miR-19b-3p	SOCS3
hsa_circ_0000563	hsa-miR-203a-3p	SOCS3
hsa_circ_0001023	hsa-miR-203a-3p	SOCS3
hsa_circ_0003322	hsa-miR-181a-5p	ZNF527
hsa_circ_0003322	hsa-miR-181b-5p	ZNF527
hsa_circ_0003322	hsa-miR-181c-5p	ZNF527
hsa_circ_0003972	hsa-miR-203a-3p	SOCS3
hsa_circ_0004212	hsa-miR-34a-5p	HSPA1B
hsa_circ_0005309	hsa-miR-181c-5p	ZNF527
hsa_circ_0005309	hsa-miR-34a-5p	HSPA1B
hsa_circ_0006607	hsa-miR-203a-3p	SOCS3
hsa_circ_0008510	hsa-miR-34a-5p	HSPA1B
hsa_circ_0008903	hsa-miR-26a-5p	HSPA8
hsa_circ_0008903	hsa-miR-26b-5p	HSPA8
hsa_circ_0039927	hsa-miR-203a-3p	SOCS3
hsa_circ_0085440	hsa-miR-181a-5p	ZNF527
hsa_circ_0085440	hsa-miR-181b-5p	ZNF527
hsa_circ_0085440	hsa-miR-181c-5p	ZNF527
hsa_circ_0108796	hsa-miR-140-5p	EGR2
hsa_circ_0108796	hsa-miR-26a-5p	HSPA8
hsa_circ_0108796	hsa-miR-26b-5p	HSPA8
hsa_circ_0108796	hsa-miR-34a-5p	HSPA1B
hsa_circ_0109744	hsa-miR-130a-3p	HSPA8
hsa_circ_0109744	hsa-miR-19a-3p	SOCS3
hsa_circ_0109744	hsa-miR-19b-3p	SOCS3
hsa_circ_0109744	hsa-miR-26a-5p	HSPA8
hsa_circ_0109744	hsa-miR-26b-5p	HSPA8
hsa_circ_0109744	hsa-miR-34a-5p	HSPA1B
circRNA8848	hsa-miR-93-5p	HSPA8

Table S4. Summary of the diverse functional roles of the seven identified mRNAs in virus-host interactions.

Gene	Associated Virus	Summary of Mechanisms
HSPA8	PRRSV, JEV, HBV	1. Interacts with PRRSV GP4 to mediate viral attachment and internalization.<br>2. Enhances PRRSV replication by facilitating RAB18/PLIN2 interaction via chaperone-mediated

Gene	Associated Virus	Summary of Mechanisms
		autophagy (CMA).<br>3. Involved in viral RNA release during JEV infection.<br>4. HSPA8 displays dual functions in HBV-infected hepatocellular carcinomas by upregulating replication and iron death.
HSPA1B	ECTV, ORFV	1. Is required for efficient replication of ECTV.<br>2. Inhibits viral proliferation during the mid-to-late stages of ORFV infection.
EGR2	Influenza Virus,	1.Functions as a transcription factor; its deficiency significantly reduces host resistance to influenza virus , attenuating the antiviral response.
SOCS3	HSV-1, Influenza A, HIV-1, PRRSV	1. HSV-1 up-regulates SOCS3 through STAT3 activation to inhibit JAK/STAT antiviral signaling<br>2. Influenza A virus weakens type I interferon response by inducing SOCS3 through an NF-κB-dependent pathway<br>3. HIV-1 instead triggers sustained immune activation through down-regulation of SOCS3 triggers sustained immune activation and promotes viral replication<br>4. PRRSV can promote viral replication by inducing SOCS3 expression through the p38/AP-1 signaling pathway
ZNF527	TGEV,SARS-CoV-2	1.During TGEV infection,ZNF268a may play a potential role in antiviral immunity by promoting NF-κB signaling through maintaining IKK complex stability<br>2.Zinc finger protein family members can promote the abundance and antiviral activity of immune cells, suggesting a potential role in inhibiting SARS-CoV-2 infection.
CXCR4	HIV-1, HSV-1, EBV,	1. Serves as a key co-receptor for HIV-1 entry into CD4+ T cells.<br>2. Downregulated by HSV-1 and EBV to impair immune cell trafficking and function, facilitating viral immune escape.
NOTCH3	HIV, SARS-CoV-2, HCV	1. HIV Tat protein drives neuroinflammation by enhancing NOTCH3 signaling<br>2. Alveolar foci are accompanied by NOTCH3 up-regulation in SARS-CoV-2 infections<br>3. HCV NS3 protein promotes persistent viral infection by modulating SRCAP/p400 activation of the Notch pathway
